# Diagnostic difficulty identifying *Apophysomyces trapeziformis* septic arthritis in a patient with multiple myeloma

**DOI:** 10.1099/jmmcr.0.005075

**Published:** 2016-12-19

**Authors:** J. Bradford Bertumen, Wiley A. Schell, Maria Joyce, Christopher Alley, Christopher W. Woods

**Affiliations:** ^1^​ Duke University Medical Center, 2100 Erwin Road, Durham, NC 27710, USA; ^2^​ Durham VA Medical Center, 508 Fulton Street, Durham, NC 27705, USA

**Keywords:** mucormycosis, *Apophysomyces*, mould, methenamine silver staining, septic arthritis, amputation

## Abstract

**Introduction:**

Mucormycosis is a rare fungal infection, but can cause substantial morbidity and mortality in both immunocompromised and immunocompetent patients. *Apophysomyces* is a mucormycetes species ubiquitous in nature, particularly in soil, decaying wood and other organic matter. *Apophysomyces* is known to cause cutaneous fungal infections, particularly after penetrating trauma. Septic arthritis is a rare clinical manifestation.

**Case presentation:**

We describe a case of *Apophysomyces trapeziformis* causing septic arthritis of the knee of a patient with multiple myeloma. He was treated multiple times for bacterial septic arthritis with minimal improvement. Surgical tissue specimens finally grew mucoraceous mould, and DNA sequencing and morphological assessment of spores identified the mould as *A. trapeziformis*. The patient was treated with amphotericin B and posaconazole, but ultimately required an above-the-knee amputation for definitive treatment.

**Conclusion:**

This case illustrates the need to evaluate for fungal infection in a persistent septic arthritis that is culture negative and refractory to empiric antibiotics, particularly in an immunocompromised individual. It also shows the importance of a thorough social history and adequate tissue specimens for culture.

## Introduction

Mucormycosis (zygomycosis) is a rare fungal infection caused by moulds in the order Mucorales. These moulds are ubiquitous in nature and can be found in soil, decaying wood and other organic matter (Neblett Fanfair *et al.*, 2012). They can cause a variety of infections, with skin and soft tissue infections being a common clinical manifestation. These infections can be quite devastating, causing complications such as tissue necrosis and necrotizing fasciitis, especially in immunocompromised individuals (Neblett Fanfair *et al.*, 2012). There is substantial morbidity and mortality associated with these infections (Vaezi *et al.*, 2016).

*Apophysomyces* species are an unusual cause of mucormycosis, accounting for 3 % of cases reported in the literature (Echaiz *et al.*, 2013). Cases involving this mould are usually associated with penetrating trauma, particularly after natural disasters. The species *Apophysomyces trapeziformis* was implicated in cases of necrotizing soft tissue infections following the tornado that ravaged Joplin, Missouri, in 2011 and the Indian Ocean tsunami in 2004 (Gomes *et al.*, 2011; Neblett Fanfair *et al.*, 2012).

Mucoraceous moulds rarely cause septic arthritis, with only a few case reports described (Mostaza *et al.*, 1989; Parra-Ruiz *et al.*, 2008). In this report, we present a patient with multiple myeloma who presented with persistent septic arthritis of the left knee, who ultimately was found to have *A. trapeziformis* growing from his synovial cultures. This case illustrates the importance of obtaining a thorough social history and adequate tissue samples for culture, as well as considering fungal infection in cases of septic arthritis where cultures are negative for bacterial growth and empiric antibiotic therapy is ineffective.

## Case report

A 62-year-old African American male with a history of multiple myeloma previously treated with pamalidomide presented to a Veterans’ Affairs (VA) hospital with a 3 day history of progressive pain and swelling in the left knee as well as fever. X-ray of the left knee showed joint effusion. He underwent an arthroscopic incision and drainage (I&D) of the left knee, which revealed a cell count of 50 250 white blood cells (WBCs) cm^−^². Routine synovial cultures were negative for bacteria, and there were no crystals present in the fluid. The patient was started on vancomycin 1 g daily, pipercillin-tazobactam 3.375 g every 6 h and clindamycin 600 mg every 8 h prior to the procedure, and was continued on them while in the hospital. He was discharged on oral ciprofloxacin 500 mg twice a day and doxycycline 100 mg twice a day.

Five days after discharge, he presented back to the same VA hospital with recurrence of left knee pain and swelling. Repeat I&D again showed purulent fluid with a cell count of 74 663 WBCs cm^−^². Again, the synovial cultures were negative for bacteria (of note, he was started on vancomycin 1 g daily and pipercillin-tazobactam 3.375 g every 6 h prior to the procedure once again). Synovial fungal and acid fast bacilli cultures were also sent at this time, and those were negative as well. The rheumatologist also evaluated the patient, and determined that the patient did not have rheumatoid arthritis (serum rheumatoid factor and serum cyclic citrullinated peptide antibody were both negative). The patient was treated empirically for septic arthritis again, first with vancomycin and pipercillin-tazobactam, and then was discharged on intravenous antibiotics with vancomycin 1 g daily and ertapenem 1 g daily for a total of 6 weeks of therapy. The patient was also treated empirically for gout, despite no crystals, first with colchicine 0.6 mg daily for 7 days, and then with a prednisone taper (40 mg daily for 5 days, 20 mg daily for 5 days and 10 mg daily for 4 days). He followed up in an orthopaedics clinic twice, and was noted to have mild improvement in his knee pain and swelling. He reported that his symptoms mildly improved after each procedure.

Approximately 6 weeks after his discharge, the patient presented again to the VA hospital with 1 week of recurrent knee pain and swelling. Repeat I&D again revealed purulent fluid in the knee with a cell count of 155 200 WBCs cm^−^². There were atypical birefringent crystals in the fluid, with an appearance that was consistent with monosodium urate crystals.

## Diagnosis

**Fig. 1.**
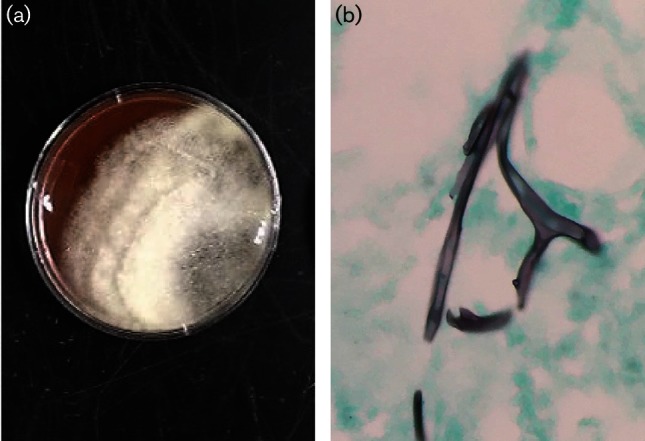
(a) Growth of *Apophysomyces trapeziformis* on synovial culture plate (sheep blood). (b) Methenamine silver stain of mucoraceous mould in tibial bone surgical specimen.

Following surgical I&D, joint tissue and synovial fluid were submitted for culture on routine microbiology media (sheep blood and chocolate agar). Overnight, the cultures revealed growth of a mould (Fig. 1). The isolate was referred to mycology for identification. Fungal synovial tissue cultures revealed moderate aseptate hyphae consistent with mucoraceous mould. The isolate was grown on potato dextrose agar (PDA) for 7 days and no sporulation was present. Approximately 0.5 cm^2^ of mycelium was bead milled for 2 min using 0.3 g of glass beads (710–1180 µm) in 700 µl of 200 mM Tris-HCl (pH 7.5), 250 mM NaCl, 25 mM EDTA and 1 % SDS. DNA was extracted using a phenol/chloroform/isoamyl alcohol procedure incorporating two 70 % ethanol wash steps. The internal transcribed spacer (ITS) region of the nuclear ribosomal DNA was amplified via PCR with the following steps: (1) 3 min at 95 °C; (2) 35 cycles of 30 s at 95 °C, 3 min at 58 °C and 1 min at 72 °C; and (3) 7 min extension at 72 °C using primer pair ITS1/ITS4. Sequencing of the amplicon (2× and 3× coverage) was performed by Eton Biosciences using primers ITS1, ITS2, ITS3, ITS4 and ApophyITS4b (5*ʹ→*3*ʹ* CTTGCTATAAACCTCGCCAAGA) (White *et al.*, 1990). The isolate was deposited with GenBank under accession no. KF 463335.1. The 777 bp sequence, corresponding to the 18S rRNA gene, ITS1, 5.8S rRNA gene, ITS2 and 28S rRNA gene, showed a 99 % match to the sequence of the ex-typus *Apophysomyces trapeziformis* isolate (GenBank accession no. NR 137034.1) over the entire 777 bp sequence.

Because no spores were produced after 1 week of incubation at 25 °C on PDA, an agar block containing the hyphae of the isolate was transferred to a plate containing 20 ml of sterile deionized water supplemented with 0.2 ml of 10 % filter-sterilized yeast extract solution, and incubated at 35 °C to induce sporulation (Padhye & Ajello, 1988). Sporulation occurred by day 7, and morphological assessment of the sporangiophores and sporangiospores also confirmed the isolate as *A. trapeziformis.* Antifungal susceptibilities were not performed.

Repeat X-ray showed worsening changes in the left knee, including joint space loss as well as osseous involvement with permeative appearance of the proximal tibia with aggressive periostitis. Magnetic resonance imaging of the left knee was consistent with septic arthritis complicated by multiple extra-articular and intramuscular abscesses, as well as air in the proximal tibia.

We obtained further history on the patient to determine the portal of entry for this mould. The patient noted that he had stepped on a nail with his left foot while living on a farm approximately 1 year before this admission. The wound healed after a few weeks.

## Treatment

The patient was started on liposomal amphotericin B (Ambisome) 300 mg daily when his synovial fungal cultures came back positive for mould. In addition to the antifungal treatment, we discussed surgical options with the patient along with the orthopaedics team. The patient was presented the choice between prolonged antifungal therapy plus extensive debridement of the left knee joint and the adjacent infected bone vs. left above-the-knee amputation followed by 2 weeks of Ambisome, then 6 weeks of posaconazole 400 mg twice a day for step-down therapy. He ultimately chose the above-the-knee amputation surgery as he did not want to deal with the subsequent debilitation of the debridement option, and tolerated the surgery well. Pathology, including methenamine silver staining, was performed on the tibial bone, which showed dense necrosis and inflammation of the tibial bone at the knee and surrounding soft tissue. There were numerous thick ribbon-like fungal forms with uncommon branching but no septa, confirming the presence of invasive mucorales osteomyelitis (Fig. 1). He was treated with the antifungal regimen previously outlined.

## Outcome and follow-up

The patient was seen twice in the infectious disease clinic. He tolerated the posaconazole therapy well and, other than phantom pain, denied any further issues with the left lower extremity. Despite recovery from his fungal septic arthritis, the patient still had progressive multiple myeloma, and he died from his disease 2 years after the mucormycosis infection.

## Discussion

*Apophysomyces* species were first isolated in 1979 from soil in northern India, and subsequently found in the United States, Australia, Central America and South America. The first case of human infection with this mould was in Arizona in 1985 (Gomes *et al.*, 2011). Penetrating trauma is a common route of infection. Despite occurring 1 year prior to presentation, we believe that it is possible our patient was infected when he stepped on a nail while living in a rural part of the country. If this history was known during his first or second presentation, the differential could have been broadened to fungal infections, and perhaps his leg could have been saved.

The most common risk factors other than penetrating trauma for *Apophysomyces* include diabetes mellitus, iron-rich states, acidaemia, immunosuppressive conditions such as organ transplantation, alcoholic cirrhosis and myelofibrosis (Gomes *et al.*, 2011; Neblett Fanfair *et al.*, 2012). Its main mechanism of pathogenesis is vascular invasion which causes thrombosis, which can lead to tissue necrosis. This can happen within days of inoculation (Gomes *et al.*, 2011).

Septic arthritis caused by mucormycetes is very unusual. In a 10 year retrospective study at a comprehensive cancer centre, there were 28 cases of fungal osteoarticular infection in patients, with 24 of those cases caused by moulds. Only five of those cases were caused by mucoraceous moulds. Of the 28 cases, only two were classified as septic arthritis (Kumashi *et al.*, 2006). There have been a few case reports of mucoraceous mould causing septic arthritis. There is one case of *Absidia corymbifera* causing septic arthritis in a patient with AIDS. The exact mechanism of inoculation was not determined (Parra-Ruiz *et al.*, 2008). There was also a case of *Cunninghamella bertholletiae* articular mucormycosis, also in a patient with AIDS. This was caused by cutaneous infection in the left thigh that infected the left knee joint via contiguous spread (Mostaza *et al.*, 1989).

Although *Apophysomyces* species are capable of infecting immunocompetent patients, immunocompromised patients like our patient are certainly at greater risk for mucormycosis infections. In a review of 929 reported cases of mucormycosis between 1940 and 2003, 154 (16.6 %) of those cases had a malignancy, and of those cases, 147 of them (95 %) had a haematological malignancy (Roden *et al.*, 2005). Furthermore, the patient’s synovial WBC count increased after his steroid taper for presumptive gout. Corticosteroid use is a risk factor for mucormycosis, and it is possible that this patient’s steroid use led to this laboratory finding (Gomes *et al.*, 2011).

The absence of growth on his two earlier synovial cultures is consistent with mucormycosis as it is tissue-invasive and difficult to recover without an adequate tissue specimen. When mould grows, it usually appears within 12–24 h. *Apophysomyces* species are frequently slow to sporulate on primary isolation media used for cultivation of filamentous fungi (Echaiz *et al.*, 2013). In addition, the hyphae of mucoraceous moulds are very delicate and the lack of septated hyphae prevents compartmentalization of the cytoplasm into cells. Therefore, agitation of tissue specimens or fluid samples, such as grinding or homogenization, may rupture the hyphae and render them non-viable, causing a false negative (Walsh *et al.*, 2012).

At the time of the case, there were only two antifungal therapies approved by the Food and Drug Administration (FDA) for treatment of mucormycosis: amphotericin B and posaconazole (Neblett Fanfair *et al.*, 2012). In this patient’s case, antifungal susceptibilities were not automatically tested, and his antifungal therapy was chosen based on previously published data on mucormycosis infections. Amphotericin B usually has the best *in vitro* activity against mucor species, although strains of *Apophysomyces elegans* have been shown to be more resistant to amphotericin, with MIC_50_ and MIC_90_ values of 2 and 4 µg ml^−1^, respectively (Chakrabarti *et al.*, 2010). Posaconazole is the first azole drug to demonstrate good mucor activity, with MICs ≤1 µg ml^−1^ (Chakrabarti *et al.*, 2010). As with most cutaneous and osteoarticular fungal infections, therapy is a combination of antifungal therapy as well as surgical debridement (Roden *et al.*, 2005). He opted for an amputation, which provided definitive source control, so ultimately antifungal susceptibilities were not requested for his isolate.

Outcomes have improved with mucormycosis infection with the advent of effective antifungal therapies and earlier diagnosis. If no therapy (antifungal or surgical) is administered, the outcome is nearly universally fatal. In a review of 929 mucormycosis cases, there was a 62.4 % survival rate among patients who received antifungal therapy alone, and a 57 % survival rate for patients who received surgery alone. Dual treatment with antifungal therapy and surgery resulted in a 70 % survival rate (Roden *et al.*, 2005). In a systematic review performed in Iran, overall mortality in 98 mucormycosis patients over 25 years was 40.8 %. Those patients who received any antifungal therapy had a survival rate of 61.0 % and those who received only surgery had a rate of 71.4 %. Those patients who received dual therapy of antifungals and surgery had a survival rate of 66.7 %. There were only four patients who received no therapy at all, and none of them survived. No patient with subcutaneous mucormycosis died (Vaezi *et al.*, 2016). These data demonstrate the important of both medical therapy and surgical debridement, but also show that even with aggressive therapy, mortality can still be high.

In conclusion, septic arthritis caused by mucormycetes is extremely rare, and we have presented here a case of *A. trapeziformis* septic arthritis. This case illustrates that clinicians should have fungal infection on their differential diagnosis if a septic arthritis patient fails to respond to empiric antibacterial treatment, especially if the patient is immunocompromised. It also shows the importance of prompt diagnosis; submission of adequate surgical tissue to recover the mould; initiation of both antifungal and surgical therapy; and a thorough social history in these patients.
